# Statistical shape modeling of the left atrium from MRI of patients with atrial fibrillation

**DOI:** 10.1186/1532-429X-13-S1-P57

**Published:** 2011-02-02

**Authors:** Christopher Gloschat, Joshua Cates, Brett Walker, Robert S MacLeod

**Affiliations:** 1 University of Utah, Salt Lake City, UT, USA

## Objective

To investigate possible clinical applications for shape variation in the treatment of atrial fibrillation, we have constructed statistical shape models from structural MRI of left atria to quantify morphological differences between atrial fibrillation patient populations.

## Background

Atrial Fibrillation (AF) is a heart condition characterized by chaotic electrical activity and irregular contractions in the atria. AF is a progressive disease with three stages of increasing fibrillation duration and frequency: paroxysmal, persistent, and permanent. Studies show that as AF progresses, there is a significant increase in left atrial (LA) volume correlated with structural and electrical remodeling of LA tissue. While volume changes have been observed, there has been little analysis of LA shape, which could provide insight into the mechanisms behind AF. Recent literature has described algorithms for modeling anatomical shape using dense sets of landmark points on surfaces derived from MRI image segmentations. In this study, we assess the feasibility of one such method, the particle-based modeling (PBM) algorithm developed by Cates, et al. at the University of Utah, for geometric modeling and statistical comparison of LA shape in AF patients.

## Methods

We segmented late gadolinium enhanced (LGE) images from four patient groups consisting of control (n=5), paroxysmal (n=6), persistent (n=6), and permanent (n=7) to isolate the LA chamber, excluding the pulmonary veins and the appendage. Using the PBM modeling algorithm, we constructed statistical shape models to perform pairwise comparison of each of the patient groups with the permanent AF group using a Hotelling T-squared test on the principal component loadings of the landmark points. For each patient group, the PBM models also described the group mean shapes and geometric variability.

## Results

We found significant differences in each AF group comparison, with p = 0.0007, 0.0084, and 0.0213 for control, paroxysmal, and persistent vs. permanent, respectively. These results are consistent with the hypothesis that AF induces morphological change, which becomes more significant as AF progresses. Empirically, our models show size changes in the atrium, but also shape changes that are independent of size.

## Conclusions

The PBM modeling algorithm is effective for quantifying morphological changes in the LA of AF patients, and has potential for discriminating between patients at various stages of AF. The availability of an image based means of evaluation of the progression of AF would allow for rapid and efficient means of monitoring patients and refining treatment options.

